# Diagnosing Fabry nephropathy: the challenge of multiple kidney disease

**DOI:** 10.1186/s12882-023-03388-8

**Published:** 2023-11-21

**Authors:** Pasquale Esposito, Carmela Caputo, Monica Repetto, Alberto Somaschini, Bellone Pietro, Paolo Colomba, Carmela Zizzo, Angelica Parodi, Valentina Zanetti, Marco Canepa, Virginia Eustachi, Francesca Sanguineri, Paola Mandich, Francesca Viazzi

**Affiliations:** 1 Department of Internal Medicine, University of Genoa, Genoa, Italy; 2 Unit of Nephrology, Dialysis, and Transplantation, IRCCS Ospedale Policlinico San Martino, Genoa, Italy; 3 Unit of Nephrology and Dialysis, Ospedale San Paolo, Savona, Italy; 4 Division of Cardiology and Cardiac Intensive Care Unit, Ospedale San Paolo, Savona, Italy; 5 Institute for Biomedical Research and Innovation (IRIB), National Research Council (CNR), Palermo, Italy; 6 Cardiovascular Disease Unit, IRCCS Ospedale Policlinico San Martino, Genoa, Italy; 7 Department of Neurosciences, Rehabilitation, Ophthalmology, Genetic and Maternal and Infantile Sciences, University of Genoa, Genoa, Italy; 8 IRCCS Ospedale Policlinico San Martino, Genoa, Italy

**Keywords:** Fabry disease, Fabry nephropathy, Kidney biopsy, Chronic kidney disease, Glomerulonephritis, ADPKD, Alpha-galactosidase A

## Abstract

Fabry disease (FD) is an X-linked inherited lysosomal disorder due to a deficiency of the enzyme alpha-galactosidase A (α-gla) due to mutations in the GLA gene. These mutations result in plasma and lysosome accumulation of glycosphingolipids, leading to progressive organ damage and reduced life expectancy. Due to the availability of specific disease-modifying treatments, proper and timely diagnosis and therapy are essential to prevent irreversible complications. However, diagnosis of FD is often delayed because of the wide clinical heterogeneity of the disease and multiple organ involvement developing in variable temporal sequences. This observation is also valid for renal involvement, which may manifest with non-specific signs, such as proteinuria and chronic kidney disease, which are also common in many other nephropathies. Moreover, an additional confounding factor is the possibility of the coexistence of FD with other kidney disorders. Thus, suspecting and diagnosing FD nephropathy in patients with signs of kidney disease may be challenging for the clinical nephrologist. Herein, also through the presentation of a unique case of co-occurrence of autosomal dominant polycystic kidney disease and FD, we review the available literature on cases of coexistence of FD and other renal diseases and discuss the implications of these conditions. Moreover, we highlight the clinical, laboratory, and histological elements that may suggest clinical suspicion and address a proper diagnosis of Fabry nephropathy.

## Introduction

Fabry disease (FD) is an X-linked inherited lysosomal disorder with an incidence of 1 in 40–60,000 new births in the male population. The disease is due to deficiency of the enzyme alpha-galactosidase A (α-gla), which results from mutations in the GLA gene. To date, more than 1000 mutations have been described, which may result in different clinical phenotypes and disease courses (including classical phenotype, late-onset FD, attenuated disease, and variants of uncertain significance) [1]. The common final effect of all these mutations is the accumulation in plasma and lysosomes of various glycosphingolipid substrates, particularly globotriaosylceramide (Gb3) and its deacylated form globotriaosylsphingosine (LysoGb3) and, to a lesser extent, galabiosylceramide (Gb2) and blood group B substances, which may lead to progressive organ damage and reduced life expectancy [2].

Fortunately, in view of the severity of the disease, specific therapies are now available. Indeed, FD can be treated with enzyme replacement therapy (ERT) using IV infusions of agalsidase alfa or beta or, in selected cases, with migalastat, an oral chaperone that increases the enzymatic activity of α-gla in patients carrying amenable mutations [3]. These treatments, the choice of which is influenced by the patient’s characteristics (gender, type of mutation, residual enzyme activity, and type of genetic variant) and clinical manifestations (symptoms and organ involvement), may improve the quality of life by reducing subjective symptoms and disease burden, even in patients with cardiac or renal involvement [4, 5]. However, the benefits of ERT seem to be dependent on the timing of diagnosis, since early initiation of therapy is associated with its long-term success, while treatments are less effective in patients with advanced organ damage [6, 7]. Thus, correct, and timely diagnosis of FD is crucial for proper management of these patients. Nevertheless, diagnostic latency is one of the most relevant culprits in the management of FD [8]. The diagnosis of FD is often delayed due to the high heterogeneity of the disease, which may present during childhood or later (because of its genetic background) with different signs and multiple organ involvement developing in variable temporal sequences [9].

This is also valid for renal involvement, which represents one of the main causes of disability and death in patients with FD. The most common renal manifestations include proteinuria, hypertension, and progressive chronic kidney disease (CKD). Although the severity and clinical impact of renal dysfunction in FD is clear, it should be recognized that these manifestations are not specific, because proteinuria, CKD, and hypertension, are hallmarks of many nephropathies, such as diabetes kidney disease and glomerulonephritis.

Furthermore, beyond the low specificity of the signs of Fabry nephropathy, a further pitfall to early suspicion and diagnosis of FD in patients with kidney alterations is the eventuality of the coexistence of FD with other nephropathies, which has been occasionally reported.

All these factors may make a diagnosis of FD challenging in a patient presenting with signs of kidney disease [10].

Herein, through the description of a unique case of concurrent autosomal dominant polycystic kidney disease (ADPKD) and FD, we review and discuss renal involvement in FD, highlighting the challenge posed by often overlooked conditions when multiple kidney diseases coexist.

Moreover, we provide suggestions on the clinical, laboratory, and histological elements that may help in improving diagnostic skills, emphasizing how awareness of the disease, a complete physical examination, and the combination of the different findings are essential to achieve proper and timely diagnosis.

## Renal involvement in FD

As for other organ manifestations of FD, renal damage may onset early or late during the history of the disease (Table 1). Considering the glomerular function, in the early phases of the disease, mild albuminuria and hyperfiltration have been reported [11]. However, glomerular filtration rate (GFR) calculation should be interpreted with caution since creatinine-based equations, commonly used for estimating GFR (eGFR), may be inaccurate. This is the reason why, while eGFR may represent a suitable initial test, expert panels recommend performing direct GFR measurements at least annually (e.g., iohexol GFR) to accurately assess the kidney function in patients with FD [5].

**Table 1:** Main clinical manifestation of Fabry nephropathy in males with classical phenotype^a^

Blood pressure	Low levels in patients with normal kidney function, higher prevalence of hypertension with increasing age and in patients with CKD (mostly for GFR < 60 ml/min/1.73 m^2^)
Kidney function	Hyperfiltration in early phases^b^, then progressive decrease in GFR, up to CKD and ESKD
Tubular manifestations	Distal renal tubular acidosis, isosthenuria, Fanconi syndrome, nephrogenic diabetes insipidus
Urinalysis	Microalbuminuria in early phases, then clinically manifest proteinuria Nephrotic syndrome in adulthood (18% of untreated patients) Hematuria
Urine Microscopy	Maltese crosses; Urinary mulberry cells; podocyturia
Ultrasound	Renal cysts (mainly parapelvic)

*Abbreviations*: *CKD* chronic kidney disease, *eGFR* glomerular filtration rate, *ESKD* end-stage kidney disease

^a^In patients with late-onset form, attenuated disease, or in females, clinical manifestations may be delayed or mild

^b^Hyperfiltration should be confirmed by measured GFR rather than creatinine-based estimation methods

Later, evaluating the natural history of the disease in a cohort of 105 untreated male patients, Branton et al. found that 82% of patients develop clinically manifest proteinuria at a mean age of 34 ± 10 years, while nephrotic proteinuria may be seen in 18% of patients, starting at the age of 40 ± 7 years [12]. High-grade proteinuria was often accompanied by CKD (defined as GFR < 60 ml/min/1.73 m2) that in 23% of the patients led to the development of end-stage kidney disease (ESKD) at a median age of 47 years (range, 21–56 years). However, these manifestations cannot be generalized because clinical presentation, and outcomes, may greatly vary according to age of onset, gender, genetic background, and residual enzyme activity [13]. Thus, for example, while blood pressure, because of autonomic dysfunction, may be low during the first phases of the disease [14], it may increase thereafter. The actual prevalence of hypertension in FD is unknown, but related factors include age, level of proteinuria, underlying proinflammatory environment, genetic factors, and kidney dysfunction [15]. In particular, a prevalence of hypertension of 80% has been found in untreated patients with GFR < 60 ml/min/1.73 m2 [16, 17].

A characteristic aspect reported in FD patients is the high incidence of renal cysts, mainly parapelvic, whose pathogenesis and clinical significance are unknown [18]. Urinalysis and urine microscopy, apart from albuminuria and proteinuria of different degrees, may show hematuria and peculiar features, such as “Maltese cross” particles seen at the polarized microscope and urinary “mulberry” cells, which are both expressions of the accumulation of Gb3 in epithelial cells [19, 20]. Moreover, in the presence of glomerular injury, podocyturia may also be detected [21]. However, these tests are of limited value because they are not pathognomonic for FD and are problematic to evaluate in routine laboratory examinations. Finally, although seen less frequently, tubular manifestations, such as distal renal tubular acidosis, isosthenuria, nephrogenic diabetes insipidus, and Fanconi syndrome, have also been documented [22–24]. Overall, it must be underlined that signs of FD nephropathy are not specific to FD and are commonly found in other pathological conditions, such as diabetic kidney disease and primary glomerulonephritis, which may coexist with FD [25].

## Clinical case

This unique case involves a 52-year-old man who underwent kidney transplantation at 35 years of age because of end-stage renal disease caused by autosomal dominant polycystic kidney disease (ADPKD). The patient’s father had renal cysts and died at the age of 60 of myocardial infarction, while one of two patient’s younger sisters presented with cortical and parapelvic renal cysts with normal kidney function.

In addition, he had paternal cousins with renal cysts. In his childhood, the patient reported suffering from burning in his feet and hands. At the age of 16 years, he was hospitalized for macrohematuria, proteinuria, and fever. At that time, immunological tests were negative, and serum creatinine was normal. Ultrasound imaging showed multiple liver cysts, and increased kidney volume with multiple bilateral parapelvic and cortical cysts. Hence, a clinical diagnosis of ADPKD was made. Subsequently, the patient developed hypertension and progressive CKD. In 2002, at the age of 33 years, maintenance hemodialysis was started and in 2004 the patient underwent kidney transplantation. There were no complications of the transplant surgery. Immunosuppressive treatment included tacrolimus, mycophenolate mofetil, and steroids. At discharge, serum creatinine was 167 µmol/L with associated proteinuria of about 200 mg/24 h. These values remained stable throughout the follow-up period, and no kidney biopsy was performed after the transplantation.

**Fig. 1:**
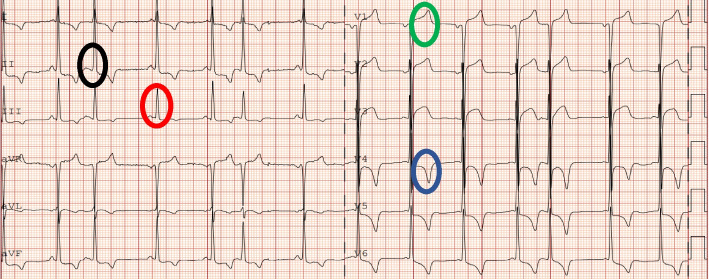
ECG showing short PR interval (108 ms) [red circle], deep negative T waves in inferior (II, III, aVF) and lateral leads (V4-V6) [blue circle], ST elevation in anterior leads (V1-V3) [green circle], and supraventricular premature beats [black circle]

At the age of 48 years, i.e., 15 years after the transplantation, the patient presented with repeated episodes of atypical chest pain. ECG showed negative T waves in the inferior and lateral leads, ST elevation in V1-V3, supraventricular premature beats, and short PR interval (Fig. 1).

**Fig. 2:**
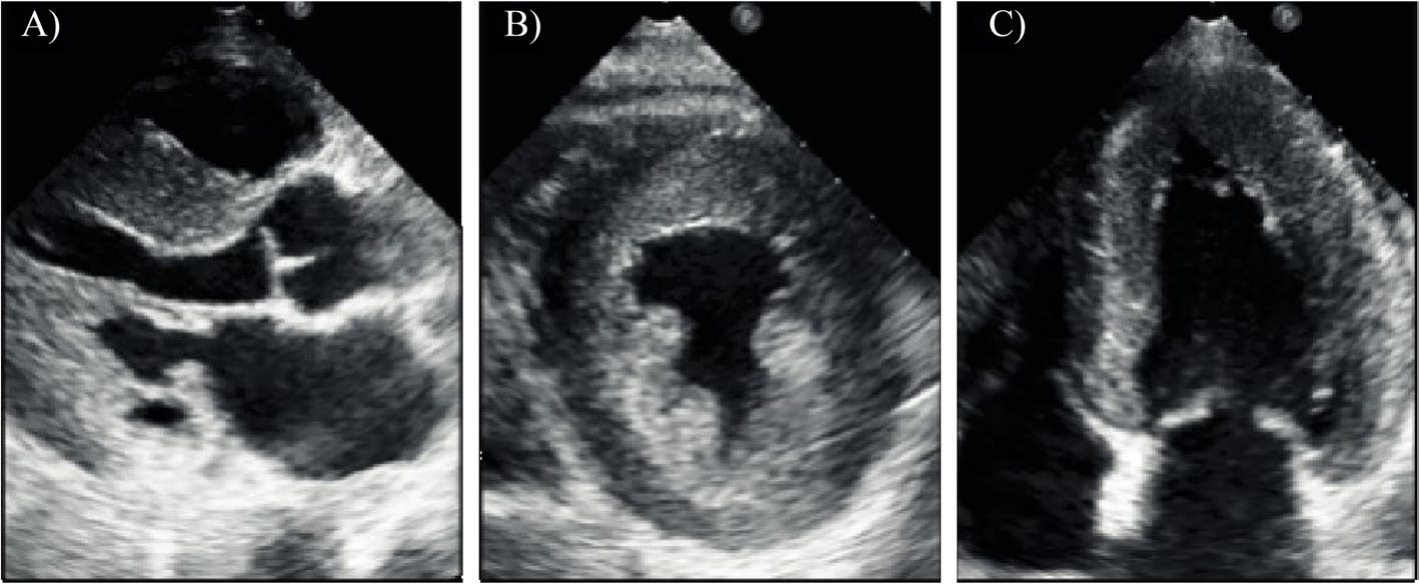
Echocardiographic views. Parasternal long-axis (**A**), parasternal short axis (**B**) and apical four chambers (**C**, frames taken during diastole). The exam revealed the presence of non-dilated left ventricle affected by marked concentric hypertrophy with ground glass appearance

The echocardiogram revealed a non-dilated left ventricle with marked concentric hypertrophy (left ventricular mass index 219.3 g/m^2^; relative wall thickness 0.837) and ground glass appearance, preserved kinesis and systolic function, advanced diastolic dysfunction with signs of elevated left ventricular filling pressures, moderate mitral regurgitation (in the presence of systolic anterior motion of the mitral valve anterior leaflet), normal right ventricle and trivial pericardial effusion) (Fig. 2).

A 24-h ECG monitoring showed rare ventricular premature beats and frequent supraventricular single premature beats with a few couples and short runs. Laboratory examinations showed that troponin and BNP values were slightly raised to 70 ng/dL (normal value < 19 ng/dL) and 250 pg/dL (normal value < 100 pg/dL), respectively. Immunofixation of serum and urine to rule out amyloidosis was negative. At that time, cardiac magnetic resonance was not performed due to concerns regarding the presence of metal plates inserted in his skull after a road accident 20 years earlier. Based on these findings, a diagnosis of hypertrophic cardiomyopathy was made. At the age of 50 years, the patient presented with a recurrence of cardiac symptoms characterized by frequent palpitations and shortness of breath. Twenty-four-hour ECG monitoring and echocardiographic findings were unchanged. In addition, for the first time, angiokeratomas on the patient’s abdomen were observed. Therefore, considering these new findings, and based on the patient’s history, enzymatic and genetic studies were performed for FD.

**Fig. 3:**
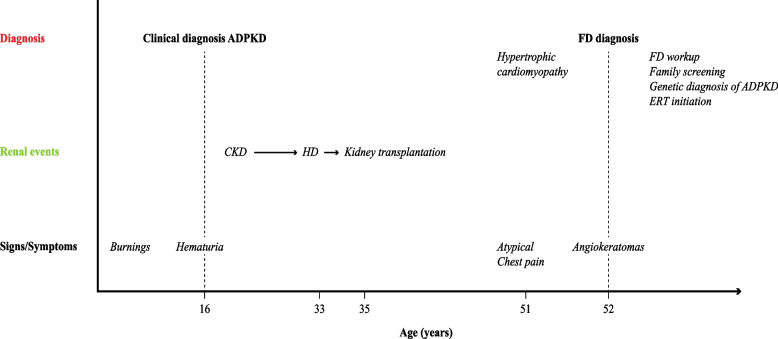
Timeline of main clinical manifestations and events of the index case. Abbreviations: ADPKD, Adult dominant polycystic kidney disease; FD, Fabry disease; CKD, chronic kidney disease; HD, hemodialysis; ERT enzyme replacement therapy

The activity of α-gla in whole blood measured by dried blood spot (DBS) was found to be extremely low (0.2 nmol/ml/h; normal range > 3) and associated with significant accumulation of LysoGb3 in plasma117.19 nmol/l (normal range < 2.3). Sanger sequencing of the *GLA* gene showed the c.1072 G > A nucleotide substitution in exon 7, which determines the amino acid substitution p. Glu358Lys. This variant is associated with the classical phenotype of FD [9]. Therefore, 16 years after kidney transplantation, the patient received a diagnosis of FD (Fig. 3).

Considering this diagnosis, cardiac and skin alterations and symptomology were retrospectively attributed to FD. As well, it was possible that the development of ESRD was due in part to FD.

To complete the clinical framework, we performed an ophthalmological examination that highlighted the presence of *cornea verticillata*. A neurological visit did not reveal symptoms attributable to peripheral neuropathy, while otolaryngological evaluation found bilateral hearing loss on high frequencies. Finally, once the compatibility of metal plates was established, brain magnetic resonance imaging did not show any abnormalities.

After completion of the diagnostic workup, the patient started ERT with algasidase beta at a dose of 1 mg/kg body weight every two weeks. Screening for FD was offered and performed on the patient’s relatives within a genetic counselling framework. Both of the patient’s sisters tested negative for the FD familial variant. However, as expected by X-linked inheritance of the disease both daughters (9 and 14 years old) were carriers and presented normal enzyme activity with low LysoGb3 accumulation (Table 2). Interestingly, the patient’s mother was negative for the c.1072 G > A substitution; thus, suggesting a de novo origin of the variant.

**Table 2:** Enzymatic and genetic testing for Fabry disease in the index case and family screening

*Patient No*	*Sex*	*Age*	*Kinship*	*Mutation in GLA gene*	*Alpha-galactosidase A activity (nmol/ml/h)* *Normal range:* > *3.0*	*LysoGb3 in plasma (nmol/l)* *Normal range:* < *2.3*
1	M	51	Index Case	p.E358K hemizygote	0.2	117.19
2	F	14	Daughter of Index Case	p.E358K heterozygote	11.9	6.74
3	F	9	Daughter of Index Case	p.E358K heterozygote	12.1	3.82
4	F	49	Sister of Index Case	wt	16.0	1.15
5	F	45	Sister of Index Case	wt	20.2	1.15
6	F	72	Mother of Index Case	wt	17.3	1.40

*Abbreviations*: *LysoGb3* globotriaosylsphingosine, *GLA* alpha-galactosidase A, *WT* wild type

Methodology: the enzyme activity of alpha-galactosidase was performed in whole blood was performed using dried blood filter paper; the determination of LysoGb3 in plasma was performed by tandem mass spectrometry (MS/MS) [26]; genetic analysis of the GLA gene was performed by Sanger sequencing

The index patient underwent also genetic testing for confirmation of ADPKD, which was considered the cause of renal dysfunction for the first 50 years of his life. Genetic testing by next-generation sequencing identified a pathogenic variant of the polycystin 2 (*PKD2*) gene. Specifically, the patient was heterozygous for the c.709 + 1G > C substitution in intron 2, resulting in a loss of the splicing site [27]. The patient’s father had died at the time of the family screening, while the mother was not tested for ADPKD because she presented normal kidney function and without renal cysts at ultrasound. Moreover, genetic testing was not performed on the patient’s daughters because they were very young and asymptomatic. Our final diagnosis was the co-occurrence of two distinct genetic diseases, namely ADPKD and FD. The former was likely inherited from the paternal line, while FD was likely due to a de novo mutation in the patient himself.

## The challenge of multiple kidney diseases

Although the present case represents one of the first descriptions of the extremely rare association of ADPKD with FD, beyond the unicity of the specific case, it allows us to make some considerations that can be generalized. First, our patient’s clinical history demonstrates that multiple unrelated inherited kidney diseases (IKDs) may coexist in the same individual with different patterns of inheritance. Apart from patients affected by a single IKD, rare cases of multiple IKDs in the same individual have been described. The concomitance of ADPKD and hereditary renal hypouricemia type 2 [28] or Alport syndrome (AS) [29] have been documented, as well as the coexistence of FD with AS [30, 31].

In the literature, there have been reports of FD coexisting with other genetic or acquired nephropathies (Table 3). Although each single case represents a rare event, together these reports emphasize that FD should not be ruled out in the diagnostic workup of patients with renal diseases. Johar et al. reported on a patient with concomitant polycystic kidney disease [32]. In addition to Fabry symptoms caused by the c.730G > A (p.Asp244Asn) mutant of the *GLA* gene that was diagnosed at the age of 34 years, this patient presented with polycystic kidney disease with multiple simple and complex cysts at 60 years of age. Molecular testing revealed a variant of unknown significance in the *PKD1* gene.

**Table 3:** Cases of kidney diseases superimposed with Fabry disease as reported in the literature

	**Cases (n)**	**Reference(s)**
**Genetic disorders**		
ADPKD	**1**^**a**^	[28]
Alport	**1**	[29]
Polycystic kidney disease	**1**	[32]
**Podocytopathies**		
MCD	**2**	[33, 34]
**Immuno-mediated diseases**		
IgAN	**4**	[35–37]
Membranous nephropathy	**3**	[38–40]
Crescentic glomerulitis	**4**	[41–43]
IgMN	**1**	[44]

*Abbreviations*: *ADPKD* autosomal dominant polycystic kidney disease, *MCD* minimal change disease, *IgAN* IgA nephropathy, *IgMN* IgM nephropathy

^a^To add the case reported in this paper

In addition to genetic disorders, FD has been reported to coexist with several types of nephropathies including crescentic glomerulonephritis, membranous nephropathy, minimal change disease (MCD), and IgA and IgM nephropathy. Singh et al. reported on two cases of necrotizing and crescentic glomerulonephritis with coexisting FD [41]. Both patients presented with fever of unknown origin and progressive renal impairment, although other pathognomic signs of FD such as dyshidrosis, acroparesthesias, and cutaneous angiokeratomas were absent. A case of crescentic glomerulonephritis in a 58-year-old woman with FD was also reported who developed progressive renal insufficiency [42]. Another case of crescentic glomerulonephritis was described in a 26-year-old woman with fever of unknown origin and renal failure [43]. Of note, the patient’s brother was also found to have FD associated with tubulointerstitial nephritis.

A few cases of superimposed FD and membranous nephropathy have been reported. Liu et al. published the case of a 21-year-old man who presented with proteinuria and stage 1 membranous nephropathy [38]. FD was diagnosed by low α-gla activity in plasma and genetic testing which revealed a hemizygous mutation in the GLA gene. In another rare case, FD was reported to coexist with membranous nephropathy in a 30-year-old male presenting with nephrotic proteinuria [39]. The diagnosis was aided by electron microscopy which showed zebra bodies in podocytes, as well as low α-gla activity and genetic testing showing a single base deletion in exon 7 of the *GLA* gene. More recently, another rare case of FD and membranous nephropathy was reported in a 22-year-old man with FD presenting with proteinuria during ERT [40]. Membranous nephropathy was confirmed by renal biopsy. Moreover, some cases of FD superimposed with MCD have been described. Even in these cases, as for membranous nephropathy, patients presented with nephrotic syndrome, suggesting that in patients with FD this condition, which is an uncommon presentation of Fabry nephropathy, deserves special attention and proper investigations [33, 34].

In addition, several cases of FD and coexisting IgA nephropathy have also been published [35]. Chao et al. presented the case of a 49-year-old man with foamy urine lasting for years [36]. Of note, the patient also reported intermittent severe burning pain in both hands during childhood. Diminished sweating and exertion were further reported along with pigmented papules in the groin area after puberty. Kidney biopsy revealed focal segmental endocapillary and mesangial proliferation with focal segmental glomerulosclerosis. Thus, the patient had many tell-tale signs including zebra bodies in podocytes under electron microscopy. Yin et al. reported on two cases of FD and IgA nephropathy [37]. Both patients presented with proteinuria and were diagnosed with IgA nephropathy upon admission with no suspicion of FD. Histology of renal biopsy showed vacuolation of podocytes with mild mesangial expansion, which raised suspicion of FD that was later confirmed by lack of α-galactosidase A activity in both patients.

Lastly, a single case of FD with coexisting IgM nephropathy has been documented [44]. The case was that of a 54-year-old woman who presented with proteinuria, but without clinical signs or family history of FD. Diagnosis of FD was obtained through the use of light and electron microscopy, immunostaining for IgM of renal biopsy, and genetic testing.

Taken together, these cases highlight that a complex clinical presentation can hide the coexistence of different diseases, even in patients with an established diagnosis. Such coexisting conditions can make diagnosis of FD even more challenging, such as in our index case who did not present with a family history of FD and had another rare disease underlying renal failure.

An open question remains the potential mutual contribution of concurrent kidney diseases on the pathogenesis and evolution of kidney dysfunction. Indeed, one can speculate that the chronic inflammatory environment present in FD may augment the risk of developing and progression of other kidney disorders [45]. For example, in our index case it is not possible to exclude that the coexistence of FD accelerated the progression of the underlying *PKD2*-related ADPKD, which typically presents with mild kidney disease [46]. Unfortunately, the limited clinical experience and the absence of mechanistic studies do not allow sound conclusions on this issue.

## When to suspect FD in patients with kidney disease

Given the unspecific signs of renal involvement in FD and the possibility of the coexistence of other kidney disorders, it seems essential for the clinical nephrologist to find elements to guide differential diagnosis and suspect FD in patients with alteration of the kidney function and/or urinalysis (mainly albuminuria). Indeed, paradoxically, due to the availability of feasible tests evaluating enzyme activity, LysoGb3 accumulation, and eventually the presence of *GLA* gene mutations, the main limitation to early diagnosis is clinical suspicion of the disease [47]. Suspicion of Fabry-related nephropathy can be guided by several aspects. Firstly, at least in males, FD should be considered in patients with (even mild) proteinuria and a more rapid loss of kidney function than the normal adult population (loss of eGFR > -1 ml/min per 1.73 m2/per year) [48]. Clinical history must be taken with special attention to family history, especially of the maternal branch, for nephropathy, kidney failure, or other alterations that could be linked to FD [49].

**Fig. 4:**
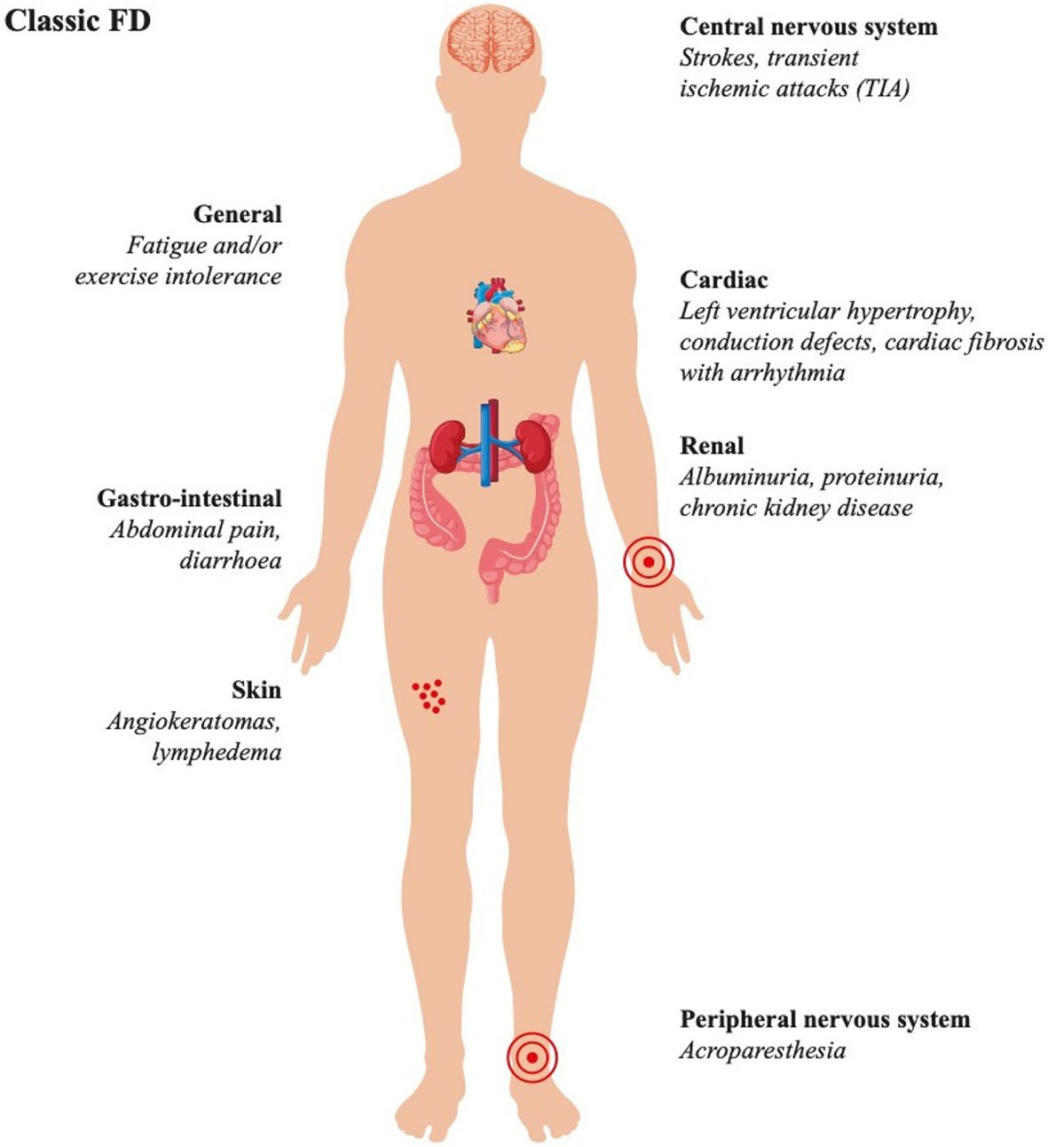
Multiorgan involvement in a classic Fabry patient. Reproduced under the terms of Creative Commons CC BY from Germain DP, Levade T, Hachulla E, Knebelmann B, Lacombe D, Seguin VL, et al. Challenging the traditional approach for interpreting genetic variants: Lessons from Fabry disease. Clin Genet. 2022;101(4):390–402. [50]

In addition, extrarenal signs and symptoms must be evaluated, starting from subjective signs, such as acroparesthesias, anhidrosis [49], history of burning or hot pain in hands and feet, and exercise, heat, or cold intolerance. Systemic organ involvement may manifest as ECG abnormalities, such as short PR interval, left ventricular hypertrophy, arrhythmia, history of early cerebrovascular disease, skin lesions (angiokeratomas), cornea verticillata, and peripheral neuropathy (Fig. 4) [50].

All these signs have been considered by some authors as “red flags” in addressing the suspicion of FD and should be taken into consideration when evaluating a patient with kidney alterations [51]. However, it is not a general rule since these signs are not so specific (for example, cornea verticillata also represents a manifestation of amiodarone-related keratopathy, or drug-induced phospholipidosis [52]). Moreover, it should be underlined that not all FD patients, including those with the classic phenotype, have complete expression of these features, and thus their absence should not exclude FD as a potential diagnosis. In addition, the extent of the disease manifestations and their temporal evolution (including kidney involvement) may vary among patients and be affected by sex, age, and classical/late-onset/attenuated disease phenotype [53]. Notably, together with the well-characterized above-reported signs, currently, it seems conceivable to suspect FD even in patients presenting with reduced kidney function and proteinuria associated with the finding of parapelvic cysts on ultrasonography [54]. Although the central role of clinical evaluation in suspecting FD, it should be recognized that, as in other nephropathies, the most reliable exam to render a diagnosis is kidney biopsy. By light microscopy, FD may present with a quite normal picture or different degrees of glomerulosclerosis, both segmental and global, interstitial fibrosis, tubular atrophy, and thickening of the vascular walls. These findings are common to most glomerular diseases, so they may be not of help in addressing clinical suspicion of FD. Instead, a peculiar aspect that can be found in the biopsy of a patient with FD is the presence of intracytoplasmic accumulation of lipids mainly in podocytes (foamy podocytes) and vacuolation in different cells [55]. These lesions are the consequence of GL3 accumulation, although they may also be present in other conditions, such as drug-related nephrotoxicity or other lysosome storage disorders [56, 57]. Notably, at light microscopy, GL3 deposits are well observable with toluidine blue staining. At immunofluorescence microscopy, nonspecific IgM or C3 deposits may be detected in areas of sclerosis. Moreover, immunofluorescence can be positive in the presence of superimposed glomerulonephritis.

Finally, electron microscopy is a fundamental tool to make a proper diagnosis of renal involvement in FD, even when other nephropathies are suspected or superimposed. Indeed, electron microscopy allows the direct identification of GL3 deposits, which may be seen in all cells, mainly podocytes and endothelial cells, as electron-dense lamellar bodies that have been described as “myelin bodies,” “onion skin,” or “zebra bodies” [58]. Lamellar bodies are cellular inclusions within lysosomes frequently appearing as intracellular concentric structures containing deposits of undegraded lipids.

However, these lesions are not specific for FD; indeed, these concentric bodies are also typical for lysosomal storage disorders (mucolipidosis type 2, GM1 gangliosidosis, Hurler’s disease, or Niemann–Pick), as well as drug-induced phospholipidosis [59]. In particular, the last condition deserves special attention in making a differential diagnosis of FD nephropathy. Drug-induced phospholipidosis is a form of acquired lysosomal storage disease characterized by intracellular accumulation of phospholipids with lamellar bodies because of the use of drugs that impair phospholipid metabolism of the lysosome [60]. These drugs include antibiotics, antidepressants, antipsychotics, antimalarials (such as chloroquine), and antiarrhythmics (such as amiodarone) [61].

This condition may represent a phenocopy of FD, especially in patients without a family history of FD, since beyond similar histological findings, patients with drug-induced phospholipidosis may present analogous clinical (including cornea verticillata) and biochemical features (such as low alpha-galactosidase activity, and elevated lysoGb3 circulating levels). In these cases, only proper genetic analysis may specifically allow FD nephropathy [62].

Finally, it should be noted that even if electron microscopy reveals diagnostic features of FD nephropathy, the limited use of this technique in clinical practice reduces its impact on the diagnosis of FD. Consequently, wider use of electron microscopy may constitute one of the factors that could facilitate the diagnostic approach to FD nephropathy.

**Fig. 5:**
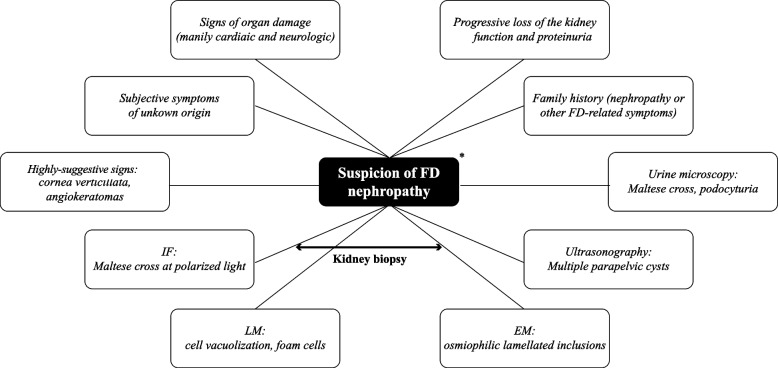
Summary of elements that may suggest a diagnosis of Fabry nephropathy in patients with kidney dysfunction and albuminuria. EM is the most important single examination leading to FD nephropathy diagnosis, beyond specific genetic and functional tests. Age of onset/severity and disease course may vary depending on gender, (better in heterozygote females than in males), residual α-gla activity and specific mutation on GLA gene. Abbreviations: LM. Light microscopy; EM, electron microscopy; IFG, immunofluorescence. *Many clinical signs and laboratory and histological features may be common to other lysosome storage disorders or drug-induced phospholipidosis

Considering all the aspects briefly discussed here, it seems clear that a suspicion of FD nephropathy in a patient with kidney disease, rather from the evidence of a single pathognomonic sign, may emerge by the combination of multiple clinical, laboratory, and histological findings (Fig. 5). Interestingly, as also demonstrated by the clinical case reported herein, such an approach could also be applied to the study of patients with ESKD.

Obviously, in this setting, urinalysis and kidney biopsy are unreliable, but accurate history (evaluating personal and familial history), as well as the assessment of multi-organ dysfunction, may equally address FD diagnosis. Once suspected, FD screening procedures include the analysis of α-gla enzyme activity in males and the search for GLA mutations in women [47]. Enzymatic activity may be tested on plasma, isolated leukocytes, or whole blood using DBS [63]. However, gene mutation analysis is mandatory for the diagnosis. Initial tests include an evaluation of plasma or urinary levels of LysoGb3, which could be useful also for monitoring the patients [64]. As a second step, systemic evaluation of organ damage should be warranted for all patients, through a complex workup, aiming to define indications, timing, modality, and monitoring of the specific treatment. The description of diagnosis and treatment strategies, such as long-term management of FD, is outside the scope of the present paper and may be found in focused expert opinions and guidelines [65].

## Conclusions

The awareness that renal signs in FD are nonspecific and of the possible coexistence of FD with other kidney diseases is extremely important since correct and timely diagnosis of FD is crucial for the proper management of these patients, also considering the availability of specific therapies. Indeed, early treatments may result in better biochemical response and slower progression of coexisting cardiac and renal disease [66–68]. An additional benefit of FD recognition is the possibility of performing family screening to allow early diagnosis of FD, thus permitting appropriate monitoring and treatment of the disease before the appearance of irreversible organ damage [69, 70]. To achieve these objectives, we need to improve diagnostic skills, first increasing understanding of the pathogenesis of FD and its presentation and considering FD in the differential diagnosis of kidney disease, even if a diagnosis is already available. Such an approach can aid in earlier diagnosis, genetic counseling, and administration of treatments that can improve long-term outcomes [71].

## Data Availability

The data underlying this article will be shared on reasonable request to the corresponding author.

## References

[1] Germain DP (2010). Fabry disease. Orphanet J Rare Dis.

[2] Desnick RJ, Ioannou YA, Scriver CR, Beaudet AL, Sly WS, Valle D (2001). α-Galactosidase a deficiency. Fabry disease. The Metabolic and Molecular Bases of Inherited Disease.

[3] Lenders M, Brand E (2021). Fabry disease: the current treatment landscape. Drugs.

[4] Lee CL, Lin SP, Niu DM, Lin HY (2022). Fabry Disease and the Effectiveness of Enzyme Replacement Therapy (ERT) in Left Ventricular Hypertrophy (LVH) improvement: a review and meta-analysis. Int J Med Sci.

[5] Ortiz A, Germain DP, Desnick RJ, Politei J, Mauer M, Burlina A (2018). Fabry disease revisited: management and treatment recommendations for adult patients. Mol Genet Metab.

[6] Banikazemi M, Bultas J, Waldek S, Wilcox WR, Whitley CB, McDonald M (2007). Agalsidase-beta therapy for advanced Fabry disease: a randomized trial. Ann Intern Med.

[7] Weidemann F, Niemann M, Breunig F, Herrmann S, Beer M, Stork S (2009). Long-term effects of enzyme replacement therapy on fabry cardiomyopathy: evidence for a better outcome with early treatment. Circulation.

[8] Reisin R, Perrin A, Garcia-Pavia P. Time delays in the diagnosis and treatment of Fabry disease. Int J Clin Pract. 2017;71(1). 10.1111/ijcp.12914 28097762

[9] Ramaswami U, Whybra C, Parini R, Pintos-Morell G, Mehta A, Sunder-Plassmann G (2006). Clinical manifestations of Fabry disease in children: data from the Fabry outcome survey. Acta Paediatr.

[10] Battaglia Y, Fiorini F, Azzini C, Esposito P, De Vito A, Granata A (2021). Deficiency in the screening process of fabry disease: analysis of chronic kidney patients not on dialysis. Front Med (Lausanne).

[11] West M, Nicholls K, Mehta A, Clarke JT, Steiner R, Beck M (2009). Agalsidase alfa and kidney dysfunction in Fabry disease. J Am Soc Nephrol.

[12] Branton MH, Schiffmann R, Sabnis SG, Murray GJ, Quirk JM, Altarescu G (2002). Natural history of Fabry renal disease: influence of alpha-galactosidase A activity and genetic mutations on clinical course. Medicine (Baltimore).

[13] Pan X, Ouyang Y, Wang Z, Ren H, Shen P, Wang W (2016). Genotype: a crucial but not unique factor affecting the clinical phenotypes in Fabry disease. PLoS One.

[14] Burlina AP, Sims KB, Politei JM, Bennett GJ, Baron R, Sommer C (2011). Early diagnosis of peripheral nervous system involvement in Fabry disease and treatment of neuropathic pain: the report of an expert panel. BMC Neurol.

[15] Del Pinto R, Ferri C (2020). The role of immunity in Fabry disease and hypertension: a review of a novel common pathway. High Blood Press Cardiovasc Prev.

[16] Kleinert J, Dehout F, Schwarting A, de Lorenzo AG, Ricci R, Kampmann C (2006). Prevalence of uncontrolled hypertension in patients with Fabry disease. Am J Hypertens.

[17] Schiffmann R, Warnock DG, Banikazemi M, Bultas J, Linthorst GE, Packman S (2009). Fabry disease: progression of nephropathy, and prevalence of cardiac and cerebrovascular events before enzyme replacement therapy. Nephrol Dial Transplant.

[18] Pisani A, PetruzzelliAnnicchiarico L, Pellegrino A, Bruzzese D, Feriozzi S, Imbriaco M (2018). Parapelvic cysts, a distinguishing feature of renal Fabry disease. Nephrol Dial Transplant.

[19] Sethuraman G, Chouhan K, Kaushal S, Sharma VK (2011). Fabry’s disease. Lancet.

[20] Shimohata H, Ogawa Y, Maruyama H, Hirayama K, Kobayashi M (2016). A renal variant of Fabry disease diagnosed by the presence of urinary mulberry cells. Intern Med.

[21] Vujkovac B, SrebotnikKirbis I, Keber T, CokanVujkovac A, Tretjak M, Rados KS (2022). Podocyturia in Fabry disease: a 10-year follow-up. Clin Kidney J.

[22] Sessa A, Meroni M, Battini G, Maglio A, Brambilla PL, Bertella M (2001). Renal pathological changes in Fabry disease. J Inherit Metab Dis..

[23] Lu Z, Wang Y, Gao L, Lin L, Hu L, Mao J (2023). Early onset of nephrogenic diabetes insipidus due to fabry disease in a child with GLA N215S mutation: Case report and literature review. Heliyon.

[24] Silva CAB, Moura-Neto JA, Dos Reis MA, Vieira Neto OM, Barreto FC (2021). Renal manifestations of Fabry disease: a narrative review. Can J Kidney Health Dis.

[25] Vujkovac B (2017). Fabry disease: diagnostic methods in nephrology practice. Clin Nephrol.

[26] Boutin M, Lavoie P, Abaoui M, Auray-Blais C (2016). Tandem mass spectrometry quantitation of lyso-Gb3 and six related analogs in plasma for Fabry disease patients. Curr Protoc Hum Genet..

[27] Richards S, Aziz N, Bale S, Bick D, Das S, Gastier-Foster J (2015). Standards and guidelines for the interpretation of sequence variants: a joint consensus recommendation of the American College of Medical Genetics and Genomics and the Association for Molecular Pathology. Genet Med.

[28] Peces R, Mena R, Peces C, Cuesta E, Selgas R, Barruz P (2020). Coexistence of autosomal dominant polycystic kidney disease type 1 and hereditary renal hypouricemia type 2: a model of early-onset and fast cyst progression. Clin Genet.

[29] Ebner K, Reintjes N, Feldkotter M, Korber F, Nagel M, Dotsch J (2017). A case report on the exceptional coincidence of two inherited renal disorders: ADPKD and Alport syndrome. Clin Nephrol.

[30] Hao W, Ao L, Zhang C, Zhu L, Xie D (2020). IgA nephropathy suspected to be combined with Fabry disease or Alport syndrome: a case report. J Int Med Res.

[31] Ren H, Li L, Yu J, Wu S, Zhou S, Zheng Y, Sun W (2019). Fabry disease and immunoglobulin A nephropathy presenting with Alport syndrome-like findings: a case report. Medicine (Baltimore).

[32] Johar L, Lee G, Martin-Rios A, Hall K, Cheng C, Lombardo D (2022). Polycystic kidney disease complicates renal pathology in a family with Fabry disease. Mol Genet Metab Rep.

[33] Salerno FR, Roggero L, Rossi F, Binaggia A, Bertoli S, Pieruzzi F (2022). Relapsing minimal change disease superimposed on late-onset p.N215S Fabry nephropathy. Clin Kidney J..

[34] Zarate YA, Patterson L, Yin H, Hopkin RJ (2010). A case of minimal change disease in a Fabry patient. Pediatr Nephrol.

[35] Yang N, Wang X, Xu F, Zeng C, Wang J, Liu Z (2017). Clinical and pathological characteristics of Fabry disease combined with IgA nephropathy in Chinese patients. Clin Nephrol..

[36] Chao CT, Lin WC, Kao TW (2012). Fabry disease and immunoglobulin A nephropathy. Nephrology (Carlton).

[37] Yin G, Wu Y, Zeng CH, Chen HP, Liu ZH (2014). Coexistence of Fabry disease and IgA nephropathy: a report of two cases. Ir J Med Sci.

[38] Liu Y, Xie H, Lin H, Chen S, Wang W, Zhao G, Zhang X (2016). Coexistence of Fabry disease and membranous nephropathy. Iran J Kidney Dis.

[39] Zhou W, Ni Z, Zhang M (2018). Hemizygous Fabry disease associated with membranous nephropathy: a rare case report. Clin Nephrol.

[40] Kanai T, Ito T, Aoyagi J, Yamagata T (2022). Membranous nephropathy without vacuolated podocytes in Fabry disease treated with agalsidase-beta and carbamazepine: a case report. Medicine (Baltimore).

[41] Singh HK, Nickeleit V, Kriegsmann J, Harris AA, Jennette JC, Mihatsch MJ (2001). Coexistence of Fabry’s disease and necrotizing and crescentic glomerulonephritis. Clin Nephrol.

[42] Shimazu K, Tomiyoshi Y, Aoki S, Sakemi T, Sugihara H (2002). Crescentic glomerulonephritis in a patient with heterozygous Fabry’s disease. Nephron.

[43] Kriegsmann J, Otto M, Wandel E, Schwarting A, Faust J, Hansen T (2003). Fabry’s disease, glomerulonephritis with crescentic and granulomatous interstitial nephritis Case of one family. Pathologe..

[44] Wu H, Behera TR, Gong J, Shen Q (2019). Coexistence of Fabry disease with IgM nephropathy: a case report. Medicine (Baltimore).

[45] Stenvinkel P, Chertow GM, Devarajan P, Levin A, Andreoli SP, Bangalore S, Warady BA (2021). Chronic inflammation in chronic kidney disease progression: role of Nrf2. Kidney Int Rep.

[46] Cornec-Le Gall E, Audrezet MP, Renaudineau E, Hourmant M, Charasse C, Michez E (2017). PKD2-related autosomal dominant polycystic kidney disease: prevalence, clinical presentation, mutation spectrum, and prognosis. Am J Kidney Dis.

[47] Vardarli I, Rischpler C, Herrmann K, Weidemann F (2020). Diagnosis and screening of patients with fabry disease. Ther Clin Risk Manag.

[48] Wanner C, Oliveira JP, Ortiz A, Mauer M, Germain DP, Linthorst GE (2010). Prognostic indicators of renal disease progression in adults with Fabry disease: natural history data from the Fabry Registry. Clin J Am Soc Nephrol.

[49] Laney DA, Bennett RL, Clarke V, Fox A, Hopkin RJ, Johnson J (2013). Fabry disease practice guidelines: recommendations of the National Society of Genetic Counselors. J Genet Couns.

[50] Germain DP, Levade T, Hachulla E, Knebelmann B, Lacombe D, Seguin VL (2022). Challenging the traditional approach for interpreting genetic variants: lessons from Fabry disease. Clin Genet.

[51] Pieroni M, Moon JC, Arbustini E, Barriales-Villa R, Camporeale A, Vujkovac AC (2021). Cardiac involvement in fabry disease: JACC review topic of the week. J Am Coll Cardiol.

[52] D'Amico DJ, Kenyon KR, Ruskin JN (1981). Amiodarone keratopathy: drug-induced lipid storage disease. Arch Ophthalmol.

[53] Guo W, Xie Y, Ji P, Li S, Cai G, Chen X (2023). The evolution of the initial manifestations and renal involvement of chinese patients with classical and late-onset Fabry disease at different sexes and ages. BMC Nephrol.

[54] Ries M, Bettis KE, Choyke P, Kopp JB, Austin HA, Brady RO, Schiffmann R (2004). Parapelvic kidney cysts: a distinguishing feature with high prevalence in Fabry disease. Kidney Int.

[55] Fogo AB, Bostad L, Svarstad E, Cook WJ, Moll S, Barbey F (2010). Scoring system for renal pathology in Fabry disease: report of the International Study Group of Fabry Nephropathy (ISGFN). Nephrol Dial Transplant.

[56] Joybari AY, Sarbaz S, Azadeh P, Mirafsharieh SA, Rahbari A, Farasatinasab M, Mokhtari M (2014). Oxaliplatin-induced renal tubular vacuolization. Ann Pharmacother.

[57] Surendran K, Vitiello SP, Pearce DA (2014). Lysosome dysfunction in the pathogenesis of kidney diseases. Pediatr Nephrol.

[58] Pisani A, Visciano B, Imbriaco M, Di Nuzzi A, Mancini A, Marchetiello C, Riccio E (2014). The kidney in Fabry’s disease. Clin Genet.

[59] Ferreira CR, Gahl WA (2017). Lysosomal storage diseases. Transl Sci Rare Dis.

[60] Shayman JA, Abe A (2013). Drug induced phospholipidosis: an acquired lysosomal storage disorder. Biochim Biophys Acta.

[61] Wu SZ, Liang X, Geng J, Zhang MB, Xie N, Su XY (2019). Hydroxychloroquine-induced renal phospholipidosis resembling Fabry disease in undifferentiated connective tissue disease: a case report. World J Clin Cases.

[62] Bracamonte ER, Kowalewska J, Starr J, Gitomer J, Alpers CE (2006). Iatrogenic phospholipidosis mimicking Fabry disease. Am J Kidney Dis.

[63] Daitx VV, Mezzalira J, Goldim MP, Coelho JC (2012). Comparison between alpha-galactosidase A activity in blood samples collected on filter paper, leukocytes and plasma. Clin Biochem.

[64] Aerts JM, Groener JE, Kuiper S, Donker-Koopman WE, Strijland A, Ottenhoff R (2008). Elevated globotriaosylsphingosine is a hallmark of Fabry disease. Proc Natl Acad Sci U S A.

[65] Eng CM, Germain DP, Banikazemi M, Warnock DG, Wanner C, Hopkin RJ (2006). Fabry disease: guidelines for the evaluation and management of multi-organ system involvement. Genet Med.

[66] Arends M, Wijburg FA, Wanner C, Vaz FM, van Kuilenburg ABP, Hughes DA (2017). Favourable effect of early versus late start of enzyme replacement therapy on plasma globotriaosylsphingosine levels in men with classical Fabry disease. Mol Genet Metab.

[67] Warnock DG, Ortiz A, Mauer M, Linthorst GE, Oliveira JP, Serra AL (2012). Renal outcomes of agalsidase beta treatment for Fabry disease: role of proteinuria and timing of treatment initiation. Nephrol Dial Transplant.

[68] van der Veen SJ, Korver S, Hirsch A, Hollak CEM, Wijburg FA, Brands MM (2022). Early start of enzyme replacement therapy in pediatric male patients with classical Fabry disease is associated with attenuated disease progression. Mol Genet Metab.

[69] Germain DP, Moiseev S, Suarez-Obando F, Al Ismaili F, Al Khawaja H, Altarescu G (2021). The benefits and challenges of family genetic testing in rare genetic diseases-lessons from Fabry disease. Mol Genet Genomic Med.

[70] Sodre LSS, Huaira R, Colugnati FAB, Carminatti M, Braga LSS, Coutinho MP, Fernandes N (2021). Screening of family members of chronic kidney disease patients with Fabry disease mutations: a very important and underrated task. J Bras Nefrol.

[71] Wanner C, Arad M, Baron R, Burlina A, Elliott PM, Feldt-Rasmussen U (2018). European expert consensus statement on therapeutic goals in Fabry disease. Mol Genet Metab.

